# Artificial intelligence in the prevention and early detection of postpartum depression: a systematic review and meta-analysis

**DOI:** 10.3389/fpsyt.2025.1734102

**Published:** 2026-01-20

**Authors:** Azahara Ruger-Navarrete, María Gómez-Ferrera, Beatriz Mérida-Yáñez, Juana María Vázquez-Lara, Juan Gómez-Salgado, Sofía García-Oliva, María Dolores Vázquez-Lara, Luciano Rodríguez-Diaz, Irene Antúnez-Calvente, Francisco Javier Fernández-Carrasco

**Affiliations:** 1 Department of Nursing, Faculty of Health Sciences of Ceuta, University of Granada, Ceuta, Spain; 2 Department of Nursing, Virgen del Rocío University Hospital, Junta de Andalucía, Servicio Andaluz de Salud, Seville, Spain; 3 Department of Sociology, Social Work and Public Health, Faculty of Labor Sciences, University of Huelva, Huelva, Spain; 4 Safety and Health Postgraduate Program, Universidad de Especialidades Espíritu Santo, Guayaquil, Ecuador; 5 Department of Nursing, Menéndez Tolosa Health Center, Junta de Andalucía, Servicio Andaluz de Salud, Algeciras, Spain; 6 Department of Nursing, Hospital Universitari Germans Trias i Pujol, Badalona, Spain

**Keywords:** artificial intelligence, early detection, healthcare technology, machine learning, maternal health, mental health, postpartum depression, predictive models

## Abstract

**Objective:**

Postpartum depression is a frequent complication after childbirth, affecting maternal health, infant development, and family well-being. This study evaluated the role of artificial intelligence (AI) in preventing and detecting postpartum depression early.

**Methods:**

A systematic search was conducted in Scopus, PubMed, Web of Science, and CINAHL for studies (2020–2025) applying AI to identify postpartum depression. PRISMA guidelines guided selection and appraisal. Two random-effects meta-analyses estimated pooled sensitivity and accuracy based on total sample size and reported metrics.

**Results:**

Of 1,857 records, 16 studies met inclusion criteria. Machine learning models (Random Forest, XGBoost, neural networks) showed greater accuracy than traditional methods. Integration of AI with medical records and social media data enabled earlier, personalized detection. Reported challenges included algorithmic bias, data privacy, and implementation barriers. Pooled sensitivity was 69% (95% CI: 55–81%; n=277,496) and accuracy 79% (95% CI: 73–85%; n=306,156).

**Conclusions:**

AI shows promise for enhancing postpartum depression detection and prevention but requires addressing ethical, technical, and educational challenges to achieve equitable clinical integration.

**Systematic review registration:**

https://www.crd.york.ac.uk/PROSPERO/view/CRD420251004175, identifier CRD420251004175.

## Introduction

Postpartum depression (PPD) is one of the most prevalent emotional disorders following childbirth, with implications for both the mother and the developmental outcomes of the infant, as well as the familial dynamics within the mother’s immediate social environment (1, 2). Although conditions such as the ‘baby blues’ (BB) are usually mild and self-limiting, they are recognized as potential precursors to more severe pathologies, including postpartum depression (PPD) and postpartum psychosis (PPP) (1). Given this situation, early detection becomes a public health imperative.

Although the term postpartum depression (PPD) is widely used in clinical practice and research, growing evidence suggests that it represents a partial and sometimes misleading label. Approximately 50% of depressive episodes diagnosed during the postpartum period have their onset during pregnancy, supporting a broader conceptualization within the perinatal (or peripartum) depression spectrum. This conceptual clarification, increasingly recognized in the literature, highlights the clinical relevance of preventive strategies and early detection during the antepartum period rather than an exclusive focus on the postpartum stage (3).

The prevalence of postpartum emotional disorders displays considerable variation on a geographical basis, depending on sociocultural and economic factors as well as the availability of healthcare services. Rogers et al. (4) demonstrate that in low- and middle-income countries, such as a significant portion of Asia, the prevalence of the condition can vary significantly from 3.5% to 63.3%, depending on the specific region. In contrast, Europe exhibits a more uniform prevalence, although it is also high, with a reported 55.2%. These disparities are associated with inequalities in access to perinatal care, stigma around mental health, and inadequate implementation of early detection protocols, which hinders timely diagnosis and effective treatment of these medical conditions. These figures underscore the necessity for preventive interventions initiated during pregnancy, particularly among women with a history of psychological issues or exposure to traumatic events (4, 5).

Postpartum emotional disorders, including anxiety, fears, and post-traumatic stress disorder (PTSD), manifest themselves through physiological and cognitive symptoms that affect maternal well-being and hinder the establishment of an emotional bond with the newborn (6, 7). Moreover, prenatal stress has been documented as a risk factor for autism spectrum disorders, attention deficit hyperactivity disorder, and psychotic disorders in children (8).

The field of artificial intelligence (AI), an interdisciplinary area of study that focuses on the development of intelligent systems capable of performing tasks that require analysis, reasoning, and autonomous decision-making, has emerged as a promising tool in the healthcare field. When applied to perinatal health, it facilitates enhanced prediction and monitoring of PPD through techniques such as machine learning (9). These technologies have proven useful not only in diagnosing PPD, but also in associated conditions such as gestational diabetes and premature birth (10).

Machine learning, a key component of AI, facilitates the analysis of substantial data sets to discern clinical patterns that are conducive to disease prediction. There are four distinct categories: supervised, unsupervised, semi-supervised, and reinforcement learning (11). These methodologies have already been employed with great success in the classification of medical images, prediction of clinical values, and improvement of hospital triage (12, 13).

The impact of PPD on a woman’s quality of life is notable, manifesting in feelings of worthlessness, guilt, fatigue, and breastfeeding problems. This situation has implications for the infant’s cognitive and physical development, as well as for family stability (14, 15). The implications of this dynamic extend to the father, who frequently articulates feelings of frustration and disengagement, stemming from his perceived inability to provide adequate support (16). Various biological factors (obesity, caesarean section, gestational diabetes), psychological factors (anxiety, chronic stress, low self-esteem) and social factors (insufficient support, poverty, migration) are identified as determinants of PPD (17, 18). Furthermore, obstetric violence and a lack of psychosocial preparation have been demonstrated to significantly increase the risk (19).

Healthcare professionals play a key role in the detection and monitoring of maternal emotional health issues. The utilization of tools such as the Edinburgh Postnatal Depression Scale (EPDS) facilitates the process of early screening (20). The provision of humanized and continuous care, in conjunction with health education, has been demonstrated to empower mothers and promote comprehensive recovery (21, 22).

Overall, the field of AI represents a disruptive advance in addressing postpartum emotional disorders by offering tools capable of identifying complex patterns in large volumes of data, which improves diagnostic accuracy and enables personalized interventions (5, 12). Rather than replacing traditional clinical assessment, it is more accurate to consider AI as a complement that optimizes screening and follow-up protocols, thereby promoting the integration of maternal mental health into healthcare systems (13, 23). The active involvement of healthcare professionals in the implementation and management of these technologies is vital to ensure their applicability and equity in different contexts, thereby guaranteeing comprehensive, evidence-based maternal care (2, 20).

The objective of this study was to evaluate the role of AI in the prevention and early detection of PPD.

## Methodology

### Search strategy

A systematic literature search was conducted following the criteria of the PRISMA Statement (24) (Preferred Reporting Items for Systematic reviews and Meta-Analyses), which consists of a 27-point checklist covering aspects for the preparation of a systematic review (25). Scopus, Pubmed, Web of Science (WOS) and Cumulative Index to Nursing and Allied Health Literature (CINAHL) databases were used.

The implemented protocol was duly registered in the International Prospective Register of Systematic Reviews (PROSPERO) with code CRD420251004175.

Searches were conducted using multiple combinations of controlled vocabulary and free-text terms related to artificial intelligence and postpartum depression. Specifically, the terms “Artificial Intelligence” and “Machine Learning” were searched independently and in combination with “Postpartum Depression” using Boolean AND and OR operators.

The search strategy was intentionally focused on studies explicitly addressing *postpartum depression*, in order to ensure conceptual consistency and comparability across included studies. However, it should be acknowledged that this approach may have excluded studies addressing depressive symptoms with antepartum onset or using broader terminology such as *perinatal* or *peripartum depression*, which are increasingly recognized in the literature.

The search was based on a clinically answerable question formulated using the PICO framework (26) (**Table 1**), which guided the development of the search strategy (**Table 2**). Accordingly, this systematic review aimed to answer the following research question:

**Table 1:** PICO question structure.

Patient	Women in the postpartum period.
Intervention/Exposure	Use of artificial intelligence systems for the detection and prevention of postpartum depression.
Comparison	Traditional methods of detection and prevention (such as clinical interviews or questionnaires).
Outcomes	Improvement in the early identification of postpartum depression and reduction in its prevalence.

**Table 2:** Search strategy.

Database	Search date	Search strategy	Records found
Scopus	25/01/2025	Artificial Intelligence OR Machine Learning AND Postpartum Depression	1457
Pubmed	28/01/2025	((Artificial Intelligence [MeSH Terms]) AND (Postpartum Depression [MeSH Terms])	42
	28/01/2025	(Machine Learning [MeSH Terms])) AND (Postpartum Depression [MeSH Terms])	30
WOS	28/01/2025	Artificial Intelligence AND Postpartum Depression	56
	7/02/2025	Machine Learning AND Postpartum Depression	149
CINAHL	29/01/2025	Artificial Intelligence AND Postpartum Depression	53
	7/02/2025	Machine Learning AND Postpartum Depression	68
TOTAL			1857

Record counts reflect initial database retrievals prior to merging search results and removing duplicates. Variations may occur depending on database updates and search execution parameters. In Scopus, a “search within results” approach was used to refine the initial search. Record counts therefore reflect preliminary retrievals prior to merging results and removing duplicates.

*In postpartum women, does the use of artificial intelligence–based systems, compared with traditional screening or diagnostic methods, improve the prevention and early detection of postpartum depression?*

As a result, initial record counts may vary depending on database-specific indexing, applied filters, and the date of search execution. All retrieved records were subsequently merged, and duplicates were removed prior to screening.

### Inclusion criteria and study selection

Eligibility criteria were determined on the basis of the objective of the review and in accordance with the PRISMA guidelines. Original studies published between January 2020 and February 2025, in English or Spanish, that analyzed the use of AI techniques in the prevention or early detection of postpartum depression were included. Research with experimental, analytical, or observational designs were considered eligible, provided that they directly addressed the subject under study and reported results were applicable to the human population.

Studies that were not directly related to the research objective were excluded, as were those that did not provide sufficient or extractable data for analysis. Systematic reviews, previous meta-analyses, opinion articles, letters to the editor, studies conducted on animals, and duplicate publications or those derived from the same primary data were also excluded.

The selection of studies was carried out in three phases by the authors, acting independently. Initially, the titles were subjected to review to eliminate duplicates. Subsequently, the abstracts were subjected to a process of evaluation in order to identify potentially eligible articles. These were then classified as either ‘suitable’ or ‘unsuitable’ for inclusion. In conclusion, the full texts of the articles that had been selected for review were examined to determine their inclusion in the review. The reasons for exclusion were recorded where applicable. Discrepancies were resolved by consensus, and where necessary, a third reviewer was consulted.

In order to reduce the risk of publication bias, comprehensive searches were conducted in multiple international databases. The methodological quality of the included studies was independently assessed by two reviewers using the critical appraisal tools of the Joanna Briggs Institute (JBI) (27) at the University of Adelaide. These tools facilitate the evaluation of the quality of the design, execution, and analysis of the studies, as well as the extent to which they minimize the risk of bias. Depending on the methodological design, the corresponding versions of the instruments were applied: the 8-item list for quantitative cross-sectional studies and the 11-item list for qualitative and longitudinal studies. A minimum cut-off point of 6 points was established in order to consider a study to be methodologically valid. The detailed results of these evaluations are shown in **Table 3**.

**Table 3:** Characteristics of the studies included in the review.

Author/year	Country	Type of study	Quality assessment	Abstract
Fazraningtyas et al., 2025 (28)	Indonesia	Cross-sectional observational	6/8	Ensemble learning models have been demonstrated to facilitate effective diagnosis of PPD, utilizing diverse algorithms and incorporating key PPD factors (marital status, number of living children, history of depression, fear of delivery, etc.).
Gopalakrishnan et al., 2023 (29)	India	Retrospective cohort	8/11	The Attribute Selection Hybrid Network (ASHN) hybrid model identifies signs of PPD in posts, contributing to early detection of PPD in women by analyzing the language they use on social media.
Sadjadpour et al., 2024 (30)	United States	Comparative observational	8/8	Machine learning (ML) models demonstrate equivalent efficacy to conventional methods in the early detection of depression in parents with children admitted to the Neonatal Intensive Care Unit (NICU). There is a need to implement automated strategies to enhance mental health intervention.
Park et al., 2021 (31)	United States	Retrospective observational	11/11	PPD prediction models may produce bias due to inequalities in the underlying data. Machine learning models can lead to racially biased results, and methods to reduce these biases are being evaluated.
Matsumura et al., 2025 (32)	Japan	Longitudinal cohort	11/11	Decision trees represent a highly effective tool for predicting chronic PPD, with the EPDS being also employed in this process. Despite the complexity of implementation inherent in most AI models, a simple yet effective model that can be applied in different contexts has been successfully developed.
Srivatsav & Nanthini, 2024 (33)	India	Cross-sectional observational	8/8	The LSTM-CNN model demonstrated higher accuracy in detecting PPD compared to conventional methods. This model is also useful for monitoring the symptoms of women with PPD, thereby eliminating irrelevant information in the detection process.
Shivaprasad et al., 2024 (34)	India	Cross-sectional observational	8/8	The ‘XAI’ model has proven effective in detecting PPD with a high degree of accuracy, and can be integrated into healthcare settings to support clinical decision-making by healthcare professionals.
Zhang et al., 2020 (35)	China	Prospective cohort	9/11	The ‘FFS-RF’ algorithm proves useful in reducing the number of variables without compromising accuracy, while ‘SMV’ is effective in analyzing small sample data sets, thereby reducing the risk of overfitting.
Shin et al., 2020 (36)	United States	Retrospective cohort	9/11	The combination of over-sampling techniques and imbalanced data reduction improved the accuracy of the model. It is concluded that ML can be a viable and accurate tool for the early prediction of PPD.
Tang et al., 2024 (37)	Suecia	Retrospective observational	6/11	The model, which is based on an artificial neural network, has proven to be more accurate than conventional methods, thereby reducing bias. Similarly, this model addresses the problem of imbalanced dataset characteristics in high-risk populations.
Fanos et al., 2023 (38)	Italia	Cross-sectional observational	6/8	The ‘Talking About’ algorithm relies on emotion recognition by analyzing women’s voices, thus avoiding the bias inherent in conventional questionnaires. It classifies emotions as positive or negative.
Wakefield & Frasch, 2023 (39)	United States	Retrospective observational cohort	11/11	Various databases were used to assess whether patients had been treated for PPD. Also, it is highlighted that the strongest predictor of PPD was whether the woman had suffered from depression before pregnancy, in addition to sociodemographic factors.
Andersson et al., 2021 (40)	Sweden	Prospective cohort	11/11	The data were collected and machine learning models were compared. It was observed that while women’s personality traits do exert an influence, this influence is less pronounced than that of clinical history or sociodemographic factors. The models in question have proven to be highly accurate, sensitive, and specific.
Gopalakrishnan et al., 2022 (41)	Australia	Prospective cohort	11/11	The identification of risk factors was based on data from psychometric questionnaires. These risk factors included sociodemographic factors, a history of depression, and factors influencing the baby, such as weight. The clinical dataset, together with the use of AI, improved the accuracy of the model for early detection of PPD.
Y. Liu et al., 2024 (42)	United States	Retrospective observational	11/11	Following analysis of three sets of electronic medical records and risk factors, racial bias was detected by the number of false positives. To correct this bias, various adjustments were introduced, such as not applying race to the variables, in order to achieve an effective AI model.
Trifan et al., 2020 (43)	United States	Retrospective cross-sectional	1/8	The AI system detects the language used by women on social media to identify specific symptoms of women suffering from PPD. These tools utilize natural language processing techniques, a process which involves the removal of irrelevant data from the study, thereby achieving early detection of PPD.

AI, Artificial Intelligence; PPD, Postpartum Depression; SM, Social Media; ML, Machine Learning; EPDS, Edinburgh Postnatal Depression Scale; NICU, Neonatal Intensive Care Unit; LSTM-CNN, Long Short-Term Memory – Convolutional Neural Network; XAI, Explainable Artificial Intelligence; FFS-RF, Fast Feature Selection – Random Forest; SMV, Support Vector Machine.

### Data analysis

Subsequently, two random-effects meta-analyses of proportions were performed using the total sample size and the percentage of precision and sensitivity values using StatsDirect software (4.0.4 for Windows). Due to the considerable number of studies included in the analysis (n > 10), a random effects meta-analysis model was employed to calculate the mean prevalence rates and 95% confidence intervals. The heterogeneity of the included studies was assessed using the I^2^ index, while publication bias was identified through the utilization of Egger’s test.

## Results

A total of 1,857 articles were identified by searching the Scopus, Pubmed, WOS, and CINAHL databases.

After removing duplicate records (n=235), a total of 1,622 documents were obtained.

**Figure 1:**
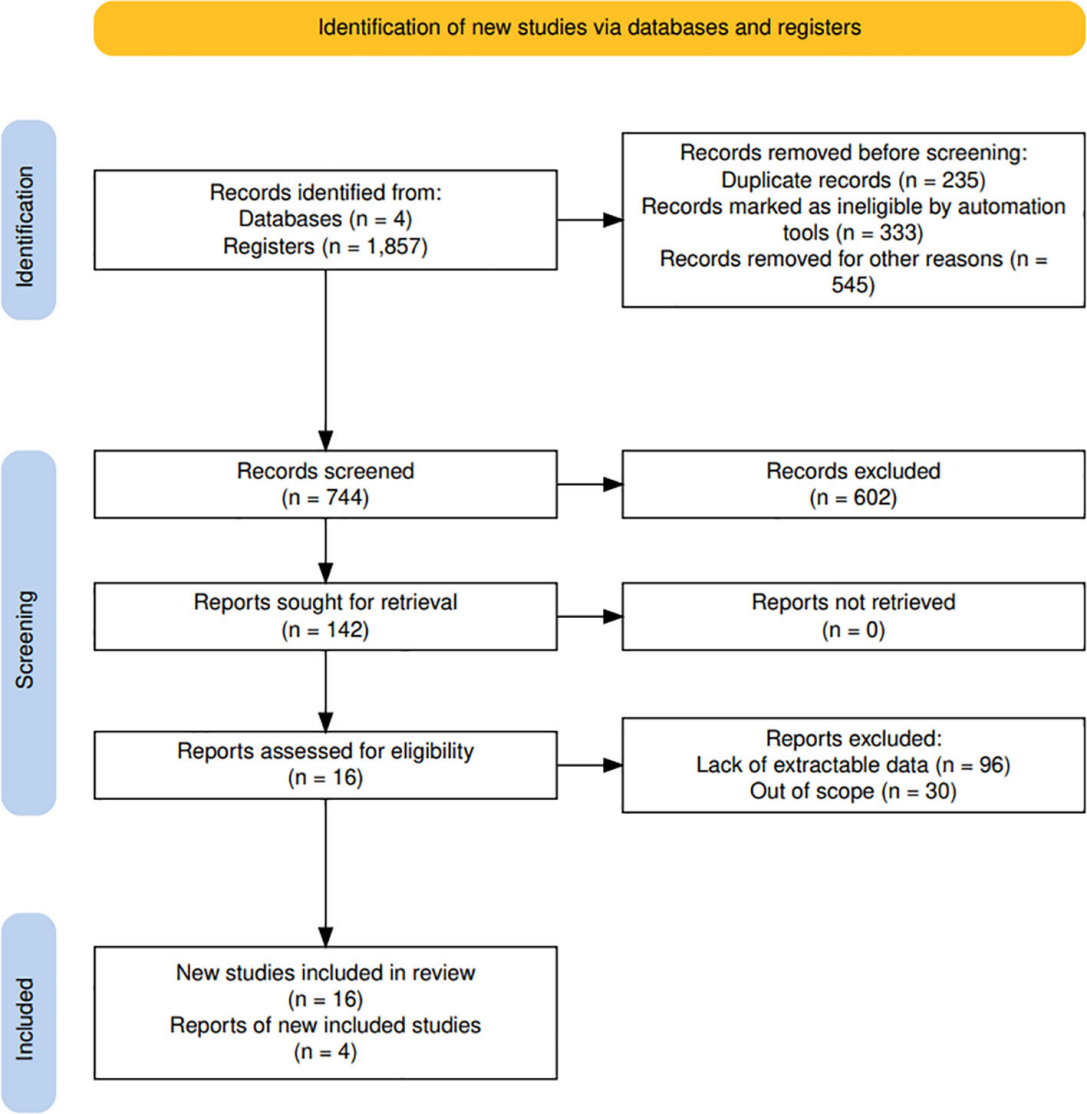
Flow chart explaining how articles were selected according to the PRISMA model.

Then, by applying the exclusion criteria and implementing the PRISMA recommendations (24), 878 documents were excluded for various reasons that failed to meet the inclusion criteria: conducted more than 5 years ago (n= 333), type of study (n= 535), language (n=10) (**Figure 1**).

Following the initial screening of the title and abstract, the number of studies was reduced from 142 to 138. This was further reduced to 16 after reading the full text, and these were finally included in the review (**Figure 1**). Of the 16 articles, 4 were cross-sectional studies, 6 were retrospective cohort studies, 4 were prospective cohort studies, 1 was a longitudinal cohort study, and 1 was a comparative observational study. Six of the articles had been conducted in the United States, three in India, two in Sweden, one in Indonesia, one in Japan, one in China, one in Australia, and one in Italy (**Table 3**).

### Puerperium and postpartum depression

Mental health often receives lower priority than reproductive health. This is particularly evident during the postpartum period, a particularly delicate period for both mother and baby (28, 33). During this time, women experience changes that make them physically and emotionally vulnerable (28). Postpartum depression is the most common complication of childbirth, with a prevalence of 10–20% (30, 32, 34–38, 40, 41).

The most common symptoms include feelings of anxiety, hopelessness, loss of self-confidence, changes in appetite, mood swings (irritation, anger, rage, or sadness), sleep disorders, and suicidal tendencies (29, 34). Additionally, these symptoms have been associated with adverse consequences, including diminished mother-infant bonding, impaired physical and cognitive development in newborns, delayed language acquisition, and altered infant behavior. In some cases, this condition has been observed to result in infanticide (33, 35, 41).

Risk factors include advanced maternal age, higher incidence in women aged 35 and over, history of psychological disorders, lack of emotional or social support, complications during childbirth, and adapting to motherhood and family pressures (29, 43).

Conversely, the manner in which a mother processes her childbirth experience, encompassing the presence of fear of delivery and the quality of sleep during pregnancy, can exert a direct influence on the onset of PPD (41). A similar pattern has been observed in women with unplanned pregnancies, premature births, or emergency C-sections, who have been found to be more susceptible to developing postpartum depression (39).

### AI in the detection and prevention of PPD

The utilization of AI has become a prominent component in the identification and mitigation of postpartum depression (34). The analysis of social media profiles serves as a predictive tool for the onset of PPD, as it has been observed that there exists a prevalence of shared emotional and linguistic patterns among women who are at risk of developing PPD (28, 38).

The development of more accurate prediction models has been facilitated by the integration of electronic medical records (28, 32, 34, 36, 39, 40, 42). In a similar way, to enhance the detection of women suffering from PPD, AI algorithms have been used, supplemented with clinical data recorded prior to delivery (28, 29, 41), leading to a more accurate diagnosis for those who require preventive treatment (39). In the context of social media, this approach has been shown to be more effective in preventing the onset of PPD compared to traditional methods (29, 30).

It has also been demonstrated that the detection of depressive symptoms based on predictive models trained with data from self-administered questionnaires has proven to be more effective than conventional models (31, 34, 35), as they integrate a greater amount of clinical data (29).

Similarly, the implementation of advanced algorithms such as Random Forest and XGBoost (eXtreme Gradient Boost) has been demonstrated to enhance the accuracy of PPD prediction (36), allowing assessing each patient’s medical history and background in terms of anxiety, educational levels, depression, etc. (39). The integration of AI technologies into mobile devices and applications has had the effect of expanding access to maternal mental health screening and monitoring (32, 33).

In order to enhance applicability in clinical settings, a study has proposed a prediction system for implementation in hospitals. This system is designed to facilitate the detection and treatment of PPD by healthcare professionals, thereby enhancing efficiency (42), improving the identification of women at risk, enabling earlier and more personalized interventions, and facilitating follow-up with predictive clinical models (40).

**Figure 2:**
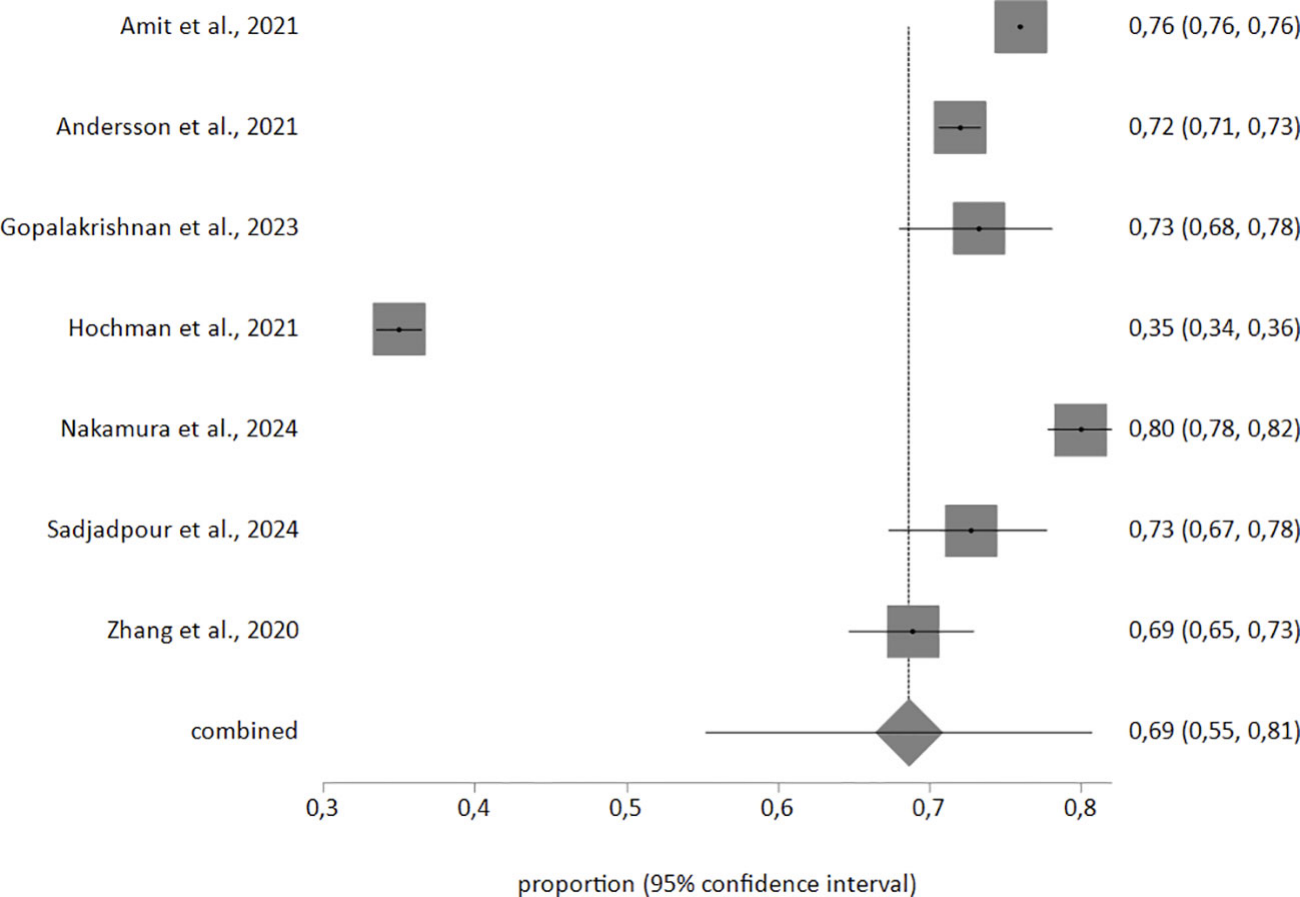
Forest plot showing sensitivity in detecting postpartum depression.

**Figure 3:**
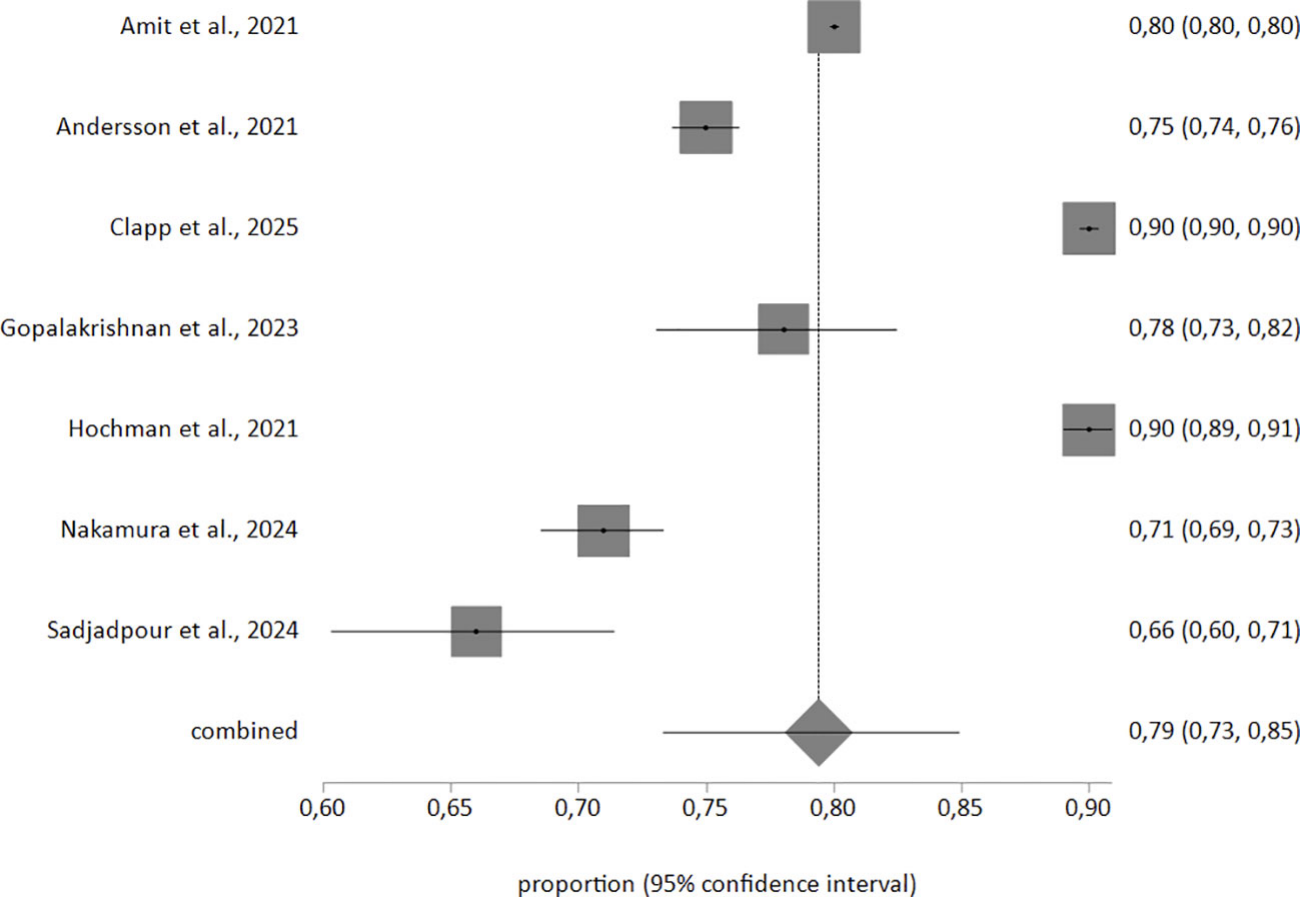
Forest plot showing accuracy in detecting postpartum depression.

In the random-effects meta-analysis of sensitivity in detecting postpartum depression, based on a sample of 277,496 individuals, sensitivity was 69% (95% confidence interval 55–81). Accuracy in detecting postpartum depression, based on a sample of 306,156 individuals, was 79% (95% confidence interval 73–85). The corresponding forest plots are shown in **Figures 2**, **3**. Heterogeneity was high (I²>90% in both meta-analyses). Egger’s test was not significant (p>0.1) in either meta-analysis.

## Discussion

The present systematic review analyses the emerging role of AI in the detection and prevention of postpartum depression, addressing whether this technology can overcome the limitations of conventional methods. The findings indicate that machine learning models, such as Random Forest and XGBoost, have demonstrated high accuracy in early identification of women at risk of PPD, with markedly improved predictive performance over conventional approaches (34–37).

Despite this, there is a lack of consensus on the efficiency of AI, underscoring the need for optimized statistical techniques such as SMOTE and Bootstrapping to enhance prediction accuracy (28). Moreover, the significance of incorporating biological and psychosocial factors in conjunction with multimodal data into predictive models is underscored to facilitate a more comprehensive perspective (33).

An additional significant aspect to consider is the applicability of AI in real-world contexts. Despite the technology’s demonstrated efficacy in clinical settings and in the analysis of electronic medical records and social media, challenges persist regarding technological infrastructure, acceptance by healthcare personnel, and ethical concerns involving data privacy and algorithmic bias (31, 37).

Finally, emphasis should be placed on the fact that AI should not replace clinical work; rather, it should act as a complementary tool that optimizes screening protocols and facilitates more personalized and effective interventions. A comprehensive approach, encompassing both parental mental health and adaptation to diverse sociocultural contexts, is crucial for the future advancement of these technologies (30, 39).

These findings should be interpreted in light of the growing recognition that postpartum depression is a misnomer, as a substantial proportion (estimated at around 50%) of depressive episodes identified during the postpartum period originate during the antepartum stage. From this perspective, the potential value of AI-based detection models extends beyond postpartum screening to earlier, preventive identification within the broader perinatal mental health continuum (3).

Notwithstanding the methodological robustness of this systematic review and meta-analysis, it is imperative to acknowledge certain limitations that may influence the interpretation of the results. Firstly, considerable methodological heterogeneity was observed among the included studies, as evidenced by the high I² values found in both meta-analyses, which exceeded 90%. This variability may be attributable to differences in populations, algorithms employed, data sources, and evaluation metrics, leading to limitations in direct comparability across studies (29, 34, 35).

It is also worthy of note that, despite the rigorous quality assessment tools (JBI) that were applied, some studies exhibited minimal acceptable scores. This may have had an impact on the robustness of the integrated evidence (42). A number of studies were identified that exhibited potential publication bias, as evidenced by the absence of negative reports or insignificant results. This bias may have led to an overestimation of the effectiveness of the analyzed models, despite the non-significance of Egger’s test.

Several studies used secondary data from social media or electronic medical records, a methodology that, while innovative, may induce selection biases and restrict the generalizability of findings to other populations (26, 27, 40). Lack of standardization in performance metrics, including sensitivity, specificity, and accuracy, further complicates quantitative and qualitative comparisons between models.

Regarding the systematic review, despite an exhaustive search being conducted in international databases, studies in languages other than English or Spanish were not included. This may have introduced a linguistic bias and limited the inclusion of relevant evidence from other regions. Finally, the exclusion of grey literature, such as theses or technical reports, may have resulted in the omission of unpublished studies that could offer valuable data in this emerging field.

## Conclusions

Recent advancements in the field of AI have led to significant progress in the prevention and detection of PPD. These developments have resulted in more accurate, personalized, and accessible diagnostic methods. The system’s capacity to analyze substantial quantities of clinical data and identify patterns on social media enables the early identification of risks, thereby facilitating timely interventions that complement conventional maternal healthcare services.

However, there are significant challenges associated with the implementation of AI, including the potential for algorithmic bias, a paucity of specific training for healthcare professionals, and ethical issues related to data protection. In order to overcome the barriers to the integration of such technologies, there is a need for an appropriate regulatory framework, awareness-raising strategies, and robust technological infrastructure.

In conclusion, the integration of AI as a support to clinical judgement, particularly in conjunction with nursing care, is essential for providing comprehensive and equitable maternal care tailored to individual needs. Future research should focus on developing models that are more explainable, accessible, and ethically responsible. Such models should be promoted to facilitate their adoption within health systems, with the aim of enhancing the quality of postpartum care.

Although this review focuses on postpartum depression, the findings should be interpreted within the broader framework of perinatal mental health. Expanding AI-based models to the antepartum period may enable earlier, preventive identification of depressive symptoms and facilitate timely implementation of psychoeducation, psychological support, structured follow-up, and referral for appropriate therapeutic interventions. Such an approach ensures that early detection translates into meaningful improvements in maternal and infant outcomes, rather than remaining a purely predictive exercise.

## Data Availability

The original contributions presented in the study are included in the article/supplementary material. Further inquiries can be directed to the corresponding author.
